# Etanercept induces remission of polyarteritis nodosa: a case report

**DOI:** 10.3389/fphar.2014.00122

**Published:** 2014-06-03

**Authors:** Maurizio Capuozzo, Alessandro Ottaiano, Eduardo Nava, Stefania Cascone, Raffaella Fico, Rosario V. Iaffaioli, Claudia Cinque

**Affiliations:** ^1^Department of Pharmacy at the Local Sanitary Agency Naples 3 SouthNaples, Italy; ^2^Department of Colorectal Oncology at the National Cancer Institute, “G. Pascale” foundationNaples, Italy; ^3^Pharmacist at the LSA Naples 1 CenterNaples, Italy

**Keywords:** polyarteritis nodosa, etanercept, glucocorticoids, immunoglobulins, thalidomide, TNF-α

Polyarteritis nodosa (PAN) is a systemic autoimmune vasculitis characterized by necrotizing inflammatory lesions of the medium-sized and small muscular arteries, preferentially at vessel bifurcations, resulting in microaneurysms formation, aneurysmal ruptures with hemorrhage, thrombosis and consequently, organs ischemia or infarction. It usually appears in middle and older age, without gender predilection (Jennette et al., 1994).

PAN shows a wide variety of symptoms, including general symptoms, neurological, skin, renal, and gastrointestinal involvement. In particular, skin lesions, characterized by multiple firm waxy papules, subcutaneous nodules, livedo reticularis, ulcers and gangrene, are observed in 25–60% of patients with PAN (Cohen et al., 1980). In most of the cases, the disease is treated with glucocorticoids and cyclosphosphamide (conventional treatment). However, rituximab is the first FDA-approved drug (on April 19, 2011) for any form of vasculitis. There is no known cause or cure for vasculitis. Rituximab works by affecting the action of and eliminating B cells, which are cells of the immune system that have a number of actions. It was initially developed for treatment of a type of lymphoma, and since has been found to be effective for autoimmune diseases, including rheumatoid arthritis and now ANCA-associated vasculitis (Cohen Tervaert, 2011).

Refractory patients are exposed to many complications, notably accelerated atherosclerosis. The etiologies of systemic vasculitis are yet unknown. However, dysregulation and/or enhanced expression of pro-inflammatory substances may be involved in the pathogenesis of these diseases. Tumor necrosis factor (TNF)-alpha is a pro-inflammatory cytokine produced primarily by cells of the macrophage-monocyte lineage. The biologic effects of TNF-alpha include adhesion molecule expression, synthesis of proinflammatory cytokines and chemokines, activation of immune system cells (T-cells, B-cells, and macrophages), and inhibition of regulatory T-cells; thus, it may directly participate in vascular inflammation as well as in endothelial cell death via apoptosis (Bansal and Houghton, 2010; Jarrot and Kaplanski, 2014). In addition, TNF-alpha may play a role in neutrophil “priming” inducing membrane expression of proteinase-3 or myeloperoxidase, which are subsequently recognized by ANCA (anti-neutrophil cytoplasmic antibodies) in ANCA-associated vasculitis (AAV) (Radford et al., 1999). Also, elevated levels of plasma TNF-alpha have been shown to correlate with active glomerulonephritis (a variant of vasculitis) and elevated in vitro TNF stimulated peripheral blood mononuclear cells and CD4+ T cells (Lúdvíksson et al., 1998).

Etanercept is a fusion protein composed of 2 extracellular p75 TNF receptor domains linked by the Fc portion of human IgG1. It binds to and neutralizes biologically active TNF-alpha. It has been approved by the FDA for the treatment of rheumatoid arthritis, polyarticular juvenile idiopathic arthritis in patients aged 2 years or older, psoriatic arthritis, ankylosing spondylitis and plaque psoriasis according to the results of randomized phase III studies. The most common side-effects of etanercept are reactions at the injection site (usually redness and sometimes itching), a blocked or runny nose, nausea, mild fever, headaches, dizziness, a rash, and stomach symptoms. More serious and infrequent adverse events are heart failure, infections and skin cancers.

In this report we show a case of successful treatment of refractory PAN with etanercept. A 10-year-old patient, with no remarkable medical history, affected by systemic vasculitis was treated with first-line glucocorticoids for 2 years. At relapse, he was treated with intravenous cyclophosphamide (IVCY). After three IVCY pulses, the patient's condition deteriorated with subacute polyarthritis, myalgias and livedo. Furthermore, multiple malacic lesions were observed at brain MRI (Magnetic Resonance Imaging) and signs of sensory-motor polyneuropathy were documented on electromyograms of the legs.

Histological examination of neuromuscular biopsy found a vasculitis with fibrinoid necrosis confirming the diagnosis of PAN. Thus, he was initially treated with 60 mg/day of prednisolone, followed by 1000 mg/day of IVCY therapy according to the European League Against Rheumatism (EULAR) recommendations for the management of small and medium vessel vasculitis (European Vasculitis Study Group, 2009). However, he presented resistance to high dose corticosteroid and IVCY therapy, thus he was treated with high dose intravenous immunoglobulin (IVIg) therapy that, according to the literature, can show efficacy in such cases with a good safety profile (Sroa et al., 2010).

After 2 months of IVIg treatment (0.4 g/kg body weight/day for 5 consecutive days every month), a partial regression of the disease was achieved. Clinical progression was observed after 6 months. Thereafter, the patient was treated with oral thalidomide (initial dosage 100 mg/day) obtaining a rapid control of signs and symptoms. The treatment was continued for 6 months with a lower daily dose (50 mg/day) until the occurrence of a severe peripheral neuropathy.

Considering (i) patient age, (ii) clinical conditions, (iii) disease progression, (iv) previous treatments, and (v) scientific literature reporting involvement of TNF pathway and response to anti-TNF agents in systemic vasculitis, off-label use of etanercept (Bartolucci et al., 2002; Sonomoto et al., 2008) was proposed (25 mg subcutaneously twice a week).

Interestingly, after starting the treatment, symptoms and lesions improved without adverse effects. A clinical response was registered after 2 months, with marked improvement in arthralgia, resolution of ulcerations and erythema nodosum and reduction of fatigue. He shows persistent clinical remission after 2 years of treatment. At present, the dosage has been adjusted according to age and weight and he is on treatment with etanercept 50 mg subcutaneously once a week.

Our report, as few cases in literature (Feinstein and Arroyo, 2005; Brik et al., 2007; Guillevin and Pagnoux, 2007; Braun-Moscovici et al., 2008; Eleftheriou et al., 2009; Valor et al., 2013; Zoshima et al., 2013), supports a role of anti-TNF therapy in rare systemic vasculitis and, indirectly, it gives evidence that increased levels of TNF-alpha may play a critical role in the inflammatory process associated with PAN. Preliminary data suggest that other several forms of vasculitis appear responsive to TNF antagonists (Behçet's disease, Churg-Strauss vasculitis and giant cell arteritis).

Despite aggressive medical management, 22.4% of patients affected by PAN die within 5 years, and of the survivors, medication-induced morbidity is frequent. Thus, there is great need of basic studies on the pathogenic mechanisms underlying these disorders as well as of treatments with high safety and efficacy. Treatment with etanercept could be considered when standard therapies are unsuccessful or contra-indicated.

## Conflict of interest statement

The authors declare that the research was conducted in the absence of any commercial or financial relationships that could be construed as a potential conflict of interest.
